# Dexamethasone and 1,25-Dihydroxyvitamin D3 Reduce Oxidative Stress-Related DNA Damage in Differentiating Osteoblasts

**DOI:** 10.3390/ijms150916649

**Published:** 2014-09-19

**Authors:** Elzbieta Pawlowska, Daniel Wysokiński, Paulina Tokarz, Agnieszka Piastowska-Ciesielska, Joanna Szczepanska, Janusz Blasiak

**Affiliations:** 1 Department of Orthodontics, Medical University of Lodz, Pomorska 251, 92-216 Lodz, Poland; E-Mail: elzbieta.pawlowska@umed.lodz.pl; 2 Department of Molecular Genetics, University of Lodz, Pomorska 141/143, 90-236 Lodz, Poland; E-Mails: dwysokinski@gmail.com (D.W.); ptokarz@biol.biol.uni.lodz.pl (P.T.); 3 Department of Comparative Endocrinology, Medical University of Lodz, Zeligowskiego 7/9, 90-752 Lodz, Poland; E-Mail: agnieszka.piastowska@umed.lodz.pl; 4 Department of Pediatric Dentistry, Medical University of Lodz, Pomorska 251, 92-216 Lodz, Poland; E-Mail: joanna.szczepanska@umed.lodz.pl

**Keywords:** oxidative stress, reactive oxygen species, osteoblast differentiation, DNA damage response, dexamethasone, 1,25-dihydroxyvitamin D3, RUNX2

## Abstract

The process of osteoblast differentiation is regulated by several factors, including RUNX2. Recent reports suggest an involvement of RUNX2 in DNA damage response (DDR), which is important due to association of differentiation with oxidative stress. In the present work we explore the influence of two RUNX2 modifiers, dexamethasone (DEX) and 1,25-dihydroxyvitamin D3 (1,25-D3), in DDR in differentiating MC3T3-E1 preosteoblasts challenged by oxidative stress. The process of differentiation was associated with reactive oxygen species (ROS) production and *tert*-butyl hydroperoxide (TBH) reduced the rate of differentiation. The activity of alkaline phosphatase (ALP), a marker of the process of osteoblasts differentiation, increased in a time-dependent manner and TBH further increased this activity. This may indicate that additional oxidative stress, induced by TBH, may accelerate the differentiation process. The cells displayed changes in the sensitivity to TBH in the course of differentiation. DEX increased ALP activity, but 1,25-D3 had no effect on it. These results suggest that DEX might stimulate the process of preosteoblasts differentiation. Finally, we observed a protective effect of DEX and 1,25-D3 against DNA damage induced by TBH, except the day 24 of differentiation, when DEX increased the extent of TBH-induced DNA damage. We conclude that oxidative stress is associated with osteoblasts differentiation and induce DDR, which may be modulated by RUNX2-modifiers, DEX and 1,25-D3.

## 1. Introduction

Osteoblast differentiation is a key process in bone formation and it is controlled by a complex signaling network with the interplay between many proteins and signaling molecules but mechanisms underlying these interactions are poorly understood [1]. The transcription factor RUNX2 (Cbfa1, Osf2) is the master regulator of this process with many partners acting downstream and upstream of it. Proteins of the SP7 and Wnt canonical signaling seems to be the most important representatives of the former and the latter, respectively [2].

Oxidative stress in the cell can be operationally defined as a state, in which the production of reactive oxygen species (ROS) exceeds the efficacy of the cellular antioxidant system. This system comprises antioxidant enzymes, DNA damage response (DDR), including DNA repair and apoptosis, and small molecular weight antioxidants, including some vitamins, reduced glutathione and other molecules [3]. Recently, autophagy was suggested to be major antioxidant mechanisms in neurodegenerative diseases [4].

Oxidative stress may induce many detrimental effects, which may contribute to age-related diseases, including osteoporosis, joint inflammatory diseases, bone tumors [5,6]. These effects are usually associated with the accumulation of oxidative stress-related damages to cellular components with age and the activation of osteoclasts by the stress resulting in increased bone resorption [7,8]. In general, oxidative stress may modulate bone metabolism by influencing the differentiation and survival of osteoblasts [9,10]. It was shown that H_2_O_2_-induced oxidative stress inhibited differentiation of mouse MC3T3-E1 pre-osteoblasts, induced antioxidant system and down-regulated genes encoding osteogenic regulators, including RUNX2 [11].

In general, ROS are linked with many pathologies, including cancer, neurodegenerative diseases, chronic inflammation, diabetes, and atherosclerosisas well as premature aging [7,12]. However, ROS may be involved in several non-devastating mechanisms, indicating that these species may play an important role in cellular signaling. ROS may induce apoptosis, but on the other hand they may regulate apoptotic processes induced by non-oxidative stimuli [13]. NADPH oxidases (Noxes) are a family of enzymes involved in physiological generation of ROS in many cells and tissues [14]. Therefore, besides their detrimental potential, ROS can be seen as indispensable cellular messenger. Their signaling role in the differentiation was appreciated in *Drosophila* hematopoietic progenitors [15] and confirmed recently in MC3T3-E1 osteoblasts [16]. Consequently, a certain level of ROS is associated with normal osteoblast differentiation, but above this level, ROS may induce harmful effects: stopping osteoblast differentiation and induce their apoptosis.

A feedback between RUNX2 and ROS may exist as over 3-fold upregulation of RUNX2 was observed in valvular interstitial cells on an incubation with hydrogen peroxide, suggesting a functional correlation between DDR and interstitial cells transdifferentiation towards an osteogenic phenotype [17]. Therefore, the maintenance of proper level of signaling ROS may be crucial for osteoblast differentiation and this level should be controlled by cellular antioxidant systems. It seems that DNA integrity should be especially aimed by these system, as DNA damage may provoke cell cycle checkpoints, which, when DNA is unrepairable, may induce apoptosis or senescence and stop differentiation. In this context, it is not surprising that RUNX2, a central regulator of osteogenesis, is involved in DDR [18]. Several lines of evidence suggest that this involvement is realized by the interaction with p53, a central regulator of DDR [19]. This is further supported by the association of changes in RUNX2 expression with numerous cancers and correlation between over-expression of RUNX2 and poor prognosis in some malignances [20,21,22]. RUNX2 may suppress apoptosis in the presence of functional p53 tumor suppressor and repress the expression of the p53-target gene, *p21^WAF1^*, which product inactivates G1/S- and S-cyclin dependent kinases, stopping the cell cycle [19,23]. This suggests a destructive role of RUNX2 in DDR, which is somehow surprising in light of the ROS production in the course of osteoblast differentiation. However, it raises a question on the significance of DNA damage in the process of osteoblast differentiation—to what extent it may be tolerated in order to safeguard the process of differentiation, otherwise stopped by apoptosis of osteoblasts? This question is especially important when the involvement of RUNX2 in tumorigenesis is taken into account.

All these data suggest that DNA damage/repair functions of RUNX2 may be important for osteoblast differentiation and this issue needs clarifying.

In the present work we investigated the extent of DNA damage induced by oxidative stress during differentiation of MC3T3-E1 osteoblasts exposed to dexamethasone and 1,25-dihydroxywitamin D3, which are recognized RUNX2 modifiers.

## 2. Results and Discussion

### 2.1. RUNX2 Inhibitors Do not Induce Changes in Morphology of Differentiating Osteoblasts

**Figure 1:**
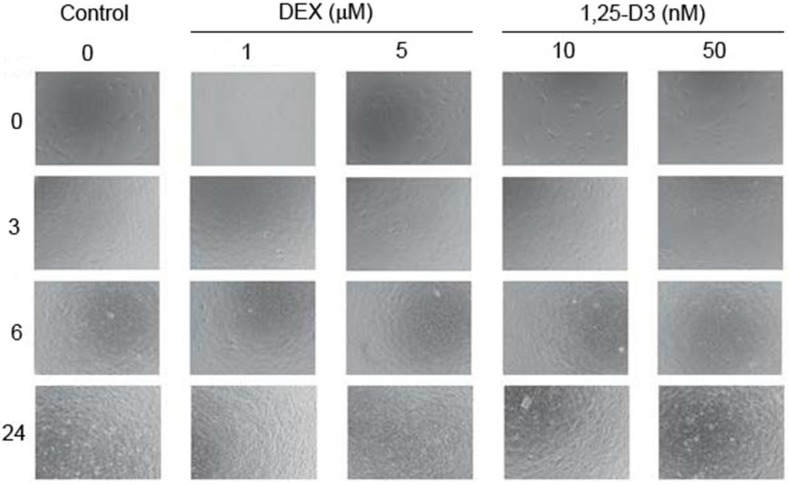
Phase-contrast micrographs of mouse MC3T3-E1 preosteoblasts treated with dexamethasone (DEX) or 1,25-dihydroxyvitamin D3 (1,25-D3) at indicated concentrations on the day 0 and 3, 6, and 24 days after induction of their differentiation as compared with untreated control cells. Images were taken at 100× magnification with Olympus CKX41 microscope (Olympus, Tokyo, Japan) equipped with Olympus DP20 digital camera.

The morphology of MC3T3-E1 cells in the course of their differentiation treated or untreated with RUNX2 activity modulators is displayed in Figure 1. Upper panel presents cells showing a typical fibroblastic morphology in logarithmic growth phase (day 0); on day 3 the cells formed a confluent monolayer with different morphology—mosaic, polygonal appearance; on days 6 and 24 mineral deposition could be noted, and confluent cells became surrounded with collagen. There was not a difference in the morphology of MC3T3-E1 cells exposed to DEX or 1,25-D3 and unexposed controls.

### 2.2. Differentiation of MC3T3-E1 Osteoblasts Is Associated with Production of Reactive Oxygen Species Potentiated by Induced Oxidative Stress

**Figure 2:**
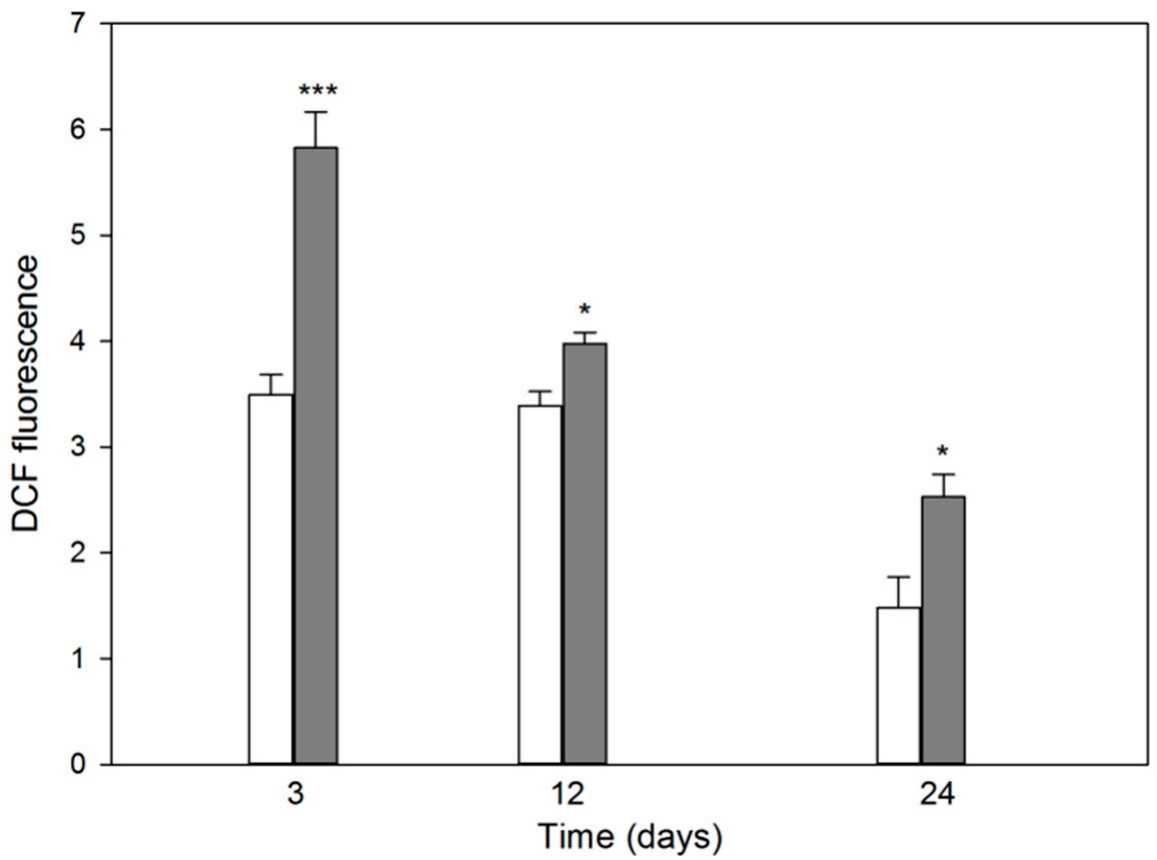
Mean intracellular reactive oxygen species (ROS) levels in differentiating mouse MC3T3-E1 preosteoblasts treated 10 min with 50 μM *tert*-butyl hydroperoxide at 37 °C (black bars) expressed by fluorescence of 2',7'-dichlorofluorescein (DCF) oxidatively converted from dichlorodihydrofluorescein diacetate as compared with untreated cells (empty bars). Error bars denote SEM, *n* = 5 for each measurement; * *p* < 0.05, *** *p* < 0.001 as compared with controls.

In the absence of TBH, intracellular ROS level, measured by 2',7'-dichlorodihydrofluorescein diacetate (DCFH-DA) fluorescence, was similar on the days 3 and 12 and decreased by about a half on day 24 (Figure 2). Cells treated by TBH displayed a significantly (*p* < 0.001) higher level of DCF-DA fluorescence than untreated cells on day 3. Although the observed difference in the level of ROS measured as DCF fluorescence on day 12 was 17% and was statistically borderline significant, we do not considered it as biologically relevant, because, as we mentioned earlier, the process of differentiation might be associated with fluctuation in ROS production. At the end of the differentiation, on day 24, this difference was significant (*p* < 0.05) and relevant (71%).

### 2.3. Oxidative Stress Stimulates Osteoblasts Differentiation

**Figure 3:**
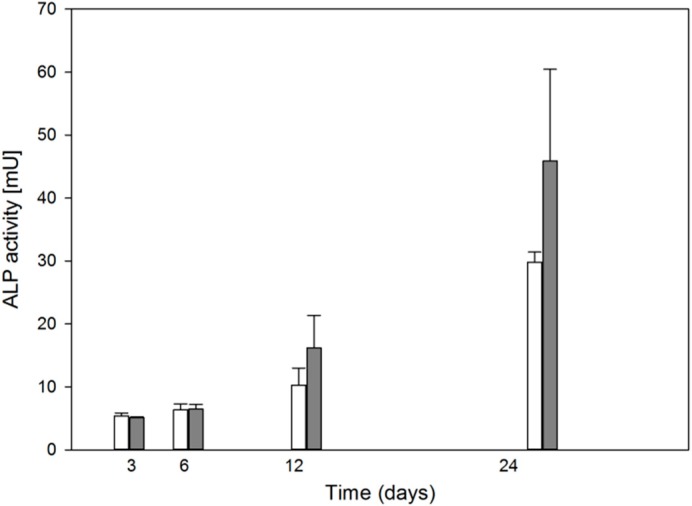
Mean activity of alkaline phosphatase (ALP) in differentiating mouse MC3T3-E1 preosteoblasts treated 10 min with 50 μM *tert*-butyl hydroperoxide at 37 °C (black bars) expressed as change in absorbance at 405 nm as compared with untreated cells (empty bars). Error bars denote SEM, *n* = 5 for each measurement.

We checked the influence of oxidative stress which was induced by TBH on the process of MC3T3-E1 preosteoblasts differentiation evaluated by the activity of alkaline phosphatase (Figure 3). We observed a monotonic increase in ALP activity in the course of the experiment. We observed also a tendency to increase the activity in the cells exposed to TBH, but this tendency was not statistically significant. However, we speculate, that it may be relevant.

### 2.4. Cells Display Different Sensitivity to DNA Damaging TBH (tert-Butyl Hydroperoxide) in the Course of Differentiation

**Figure 4:**
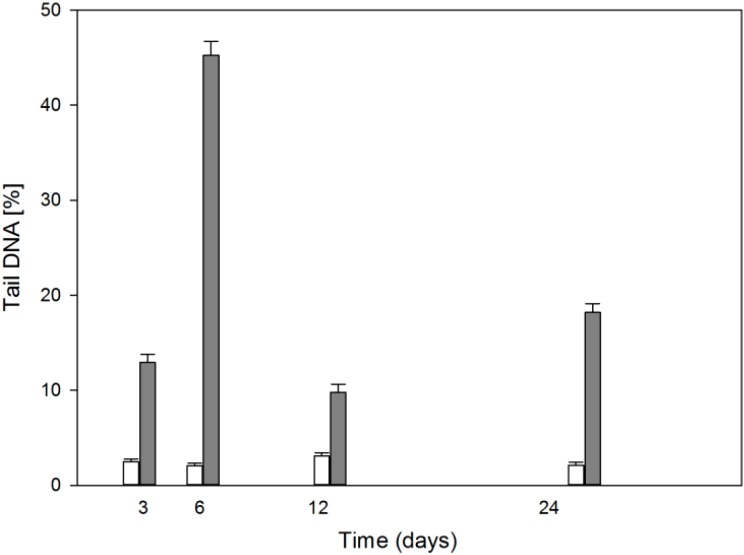
DNA damage expressed as mean percentage of DNA in the alkaline version comet tail of differentiating mouse MC3T3-E1 preosteoblasts treated 10 min with 50 μM *tert*-butyl hydroperoxide at 37 °C (black bars) as compared with untreated cells (empty bars). Error bars denote SEM, *n* = 100 in each treatment; *p* < 0.001 as compared with unexposed sample each day.

We measured the sensitivity of MC3T3-E1 cells to TBH-induced DNA damage in the course of the experiment (Figure 4). The unexposed cells displayed a small, roughly constant extent of tail DNA percentage, directly related to DNA damage. Cells exposed to 50 μM TBH showed a dramatic, from over 3 to greater than 40 times increase in the extent of damage to their DNA during osteoblast differentiation. However, this increase had its peak on day 6 and then dropped about 3 times on day 12 and increased on day 24. Qualitatively the same results were obtained for 100 μM TBH (results not shown).

### 2.5. Dexamethasone, Unlike 1,25-Dihydroxyvitamin D3, Changes the Differentiation Status of MC3T3-E1 Preosteoblasts

**Figure 5:**
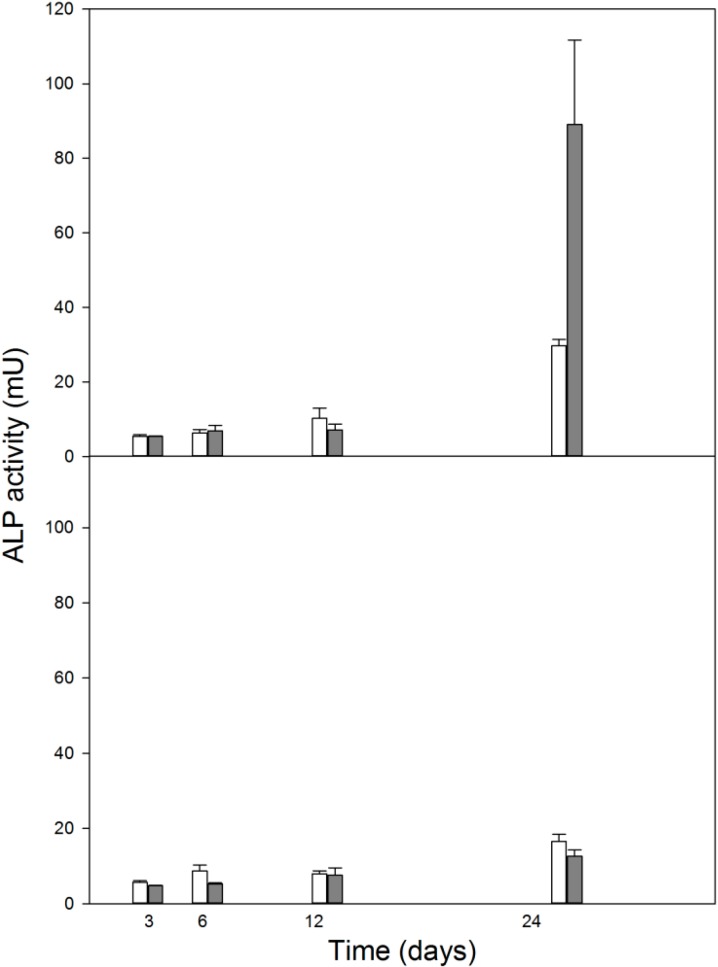
Mean activity of alkaline phosphatase (ALP) in differentiating mouse MC3T3-E1 preosteoblasts with 1 μM dexamethasone (**upper** panel, black bars) or 10 nM 1,25-dihydroxyvitamin D3 (**lower** panel, black bars) in growth medium expressed as change in absorbance at 405 nm as compared with untreated cells (empty bars). Error bars denote SEM, *n* = 5 for each measurement.

1,25-Dihydroxyvitamin D3 did not influence the differentiation status of the mouse MC3T3-E1 preosteoblasts as evaluated by the activity of alkaline phosphatase (Figure 5, lower panel). Dexomethasone neither affected this status up to day 12 of the experiment. However, we observed a dramatic increase in ALP activity on day 24 (Figure 5, upper panel), indicating a significant acceleration of the process of osteoblasts differentiation by DEX. We noted a slower rate of differentiation in experiment with 1,25-D3 than with DEX—we did not present detailed statistical analysis since this difference is obvious as it is on day 24 for DEX and lack of differences in the remaining points.

### 2.6. Dexamethasone and 1,25-Dihydroxyvitamin D3 Decrease the Extent of DNA Damage Induced by Oxidative Stress

**Figure 6:**
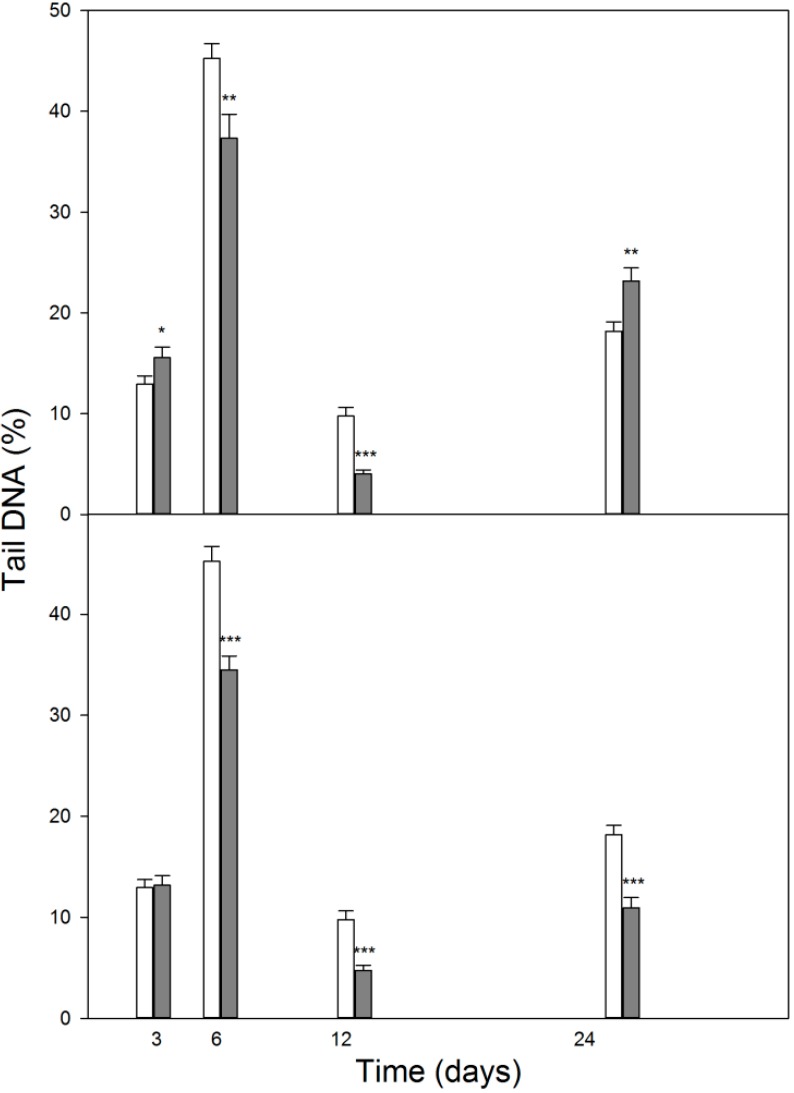
DNA damage induced by *tert*-butyl hydroperoxide (TBH) and expressed as mean percentage of DNA in tail of comets in alkaline version of the comet assay in differentiating mouse MC3T3-E1 preosteoblasts. The cells differentiate with 1 μM dexamethasone (DEX, upper panel, black bars) or 10 nM 1,25-dihydroxyvitamin D3 (1,25-D3, lower panel, black bars) in growth medium and on the indicated days they were incubated 10 min at 37 °C with 50 μM TBH and DNA damage was measured by comet assay. Error bars denote SEM, *n* = 100 in each treatment; * *p* < 0.05, ** *p* < 0.01, *** *p* < 0.001 as compared with controls containing neither DEX, nor 1,25-D3.

Neither DEX, nor 1,25-D3 affected the sensitivity of MC3T3-E1 cells to TBH up to day 3 of experiment (Figure 6). Then, we observed a significant and relevant decrease in the extent of DNA damage induced by TBH on day 6 and 12 of differentiation. This tendency was valid till the end (day 24) of observation with 1,25-D3, but reversed in the case of DEX, when we observed an increase in the extent of DNA damage induced by TBH. This effect was observed for both 10 and 50 μM TBH and 1 and 5 mM 1,25-D3.

### 2.7. Discussion

In this work, we studied some aspects of differentiation of mouse MC3T3-E1 preosteoblasts in oxidative stress and in the presence of two modulators of the activity of RUNX2, a master regulator of bone formation and protein involved in DNA damage response but by largely unknown mechanism. The results we obtained can be summarized as follows. The process of differentiation was associated with oxidative stress expressed by ROS production, which decreased at the end stage of observation (day 24, Figure 2). Additional oxidative stress generated by TBH resulted in a time-dependent reduction in ROS production. This suggests that additional mechanism(s) may be triggered by additional oxidative stress to protect differentiating osteoblasts against detrimental consequences of the stress. The activity of ALP, a marker of the process of osteoblast differentiation, increased in a time-dependent manner and TBH increased this activity on 12th and 24th days of observation (Figure 3). This may indicate that additional oxidative stress, induced by TBH, may accelerate the differentiation process. However, it is an open question whether oxidative stress itself may induce differentiation of preosteoblasts. In our experimental conditions, we did not observe typical signs of differentiation, presented in Figure 1, in cells exposed only to 50 or 100 μM TBH without adding any differentiation-inducing agent (results not shown). Next, we took a look at the extent of DNA damage in differentiating osteoblasts, both endogenous and induced by TBH (Figure 4). The cells displayed a constant level of endogenous DNA damage in the course of differentiation, but they showed fluctuation in the sensitivity to TBH, a DNA-damaging agent, in that time. Then, we included two modulators of RUNX2 activity, DEX and 1,25-D3. The latter is considered as a RUNX2 inhibitor, but there are controversies on the mechanism of action of DEX on this protein. This issue will be discussed in more detail below. First, we observed that DEX greatly increased the ALP activity on the day 24 of differentiation, but 1,25-D3 had no effect on it. This result suggests that DEX might stimulate the process of preosteoblasts differentiation. Finally, we observed a protective effect of DEX and 1,25-D3 against DNA damage induced by TBH, except the day 24 of differentiation, when DEX increased the extent of TBH-induced DNA damage (Figure 6). On the basis of the results obtained we conclude that oxidative stress is associated with osteoblasts differentiation, which was reported in previous reports, as we mentioned in the Introduction section. The role of this stress is not completely known. That is why we induced an additional stress by adding TBH to the cells, which were associated with increased ROS production. At present, it is a difficult task to assess the role of both ROS associated with the process of differentiation and these exogenous ROS, as they play an important role in the activation and inhibition of many signaling pathways and were shown to play a protective role in different forms of stress [24,25,26,27].

We observed a general increase in ALP activity during TBH treatment in the course of differentiation. Unfortunately, at present we do not have any rational explanation of this result and further mechanistic analysis is needed to clarify this effect.

We observed a decreased level of ROS produced both in TBH treated-and untreated cells in the course of their differentiation (Figure 2). This may be associated with calcification of osteoblasts and decreased absorption of external ROS, which contributes to increased DCF fluorescence primarily in TBH-treated cells.

Reactive oxygen species may induce as many as over 100 types of DNA damage, but the actual number of lesions observed in cellular DNA is lower, but this difference is likely due to technical limitations and difficulties of methods to measure the damage [28]. Although such serious DNA damages such DNA double strand breaks and inter-strand crosslinks may be induced by ROS, great majority of lesions are oxidative modifications to the DNA bases [29]. Some of such modifications may stop DNA polymerase and/or RNA polymerase, which is particularly important in differentiating cells, replicating their genomes and transcribing many genes at a high rate. The hydroxyl radical (•OH) is a major ROS effectively interacting with DNA. It mediates addition across the 5,6-pyrimidine bond and H-atom abstraction from the thymine methyl group. Cytosine and 5-methylcytosine may be affected by •OH by forming adducts, peroxyl radicals and hydroxyperoxides. The radical may be also involved in formation of 8-oxo-7,8-dihydroguanine (8-oxoG) and 2,6-diamino-4-hydroxy-5-formamidopyrimidine (Fapy-G) [29]. There are many other highly reactive ROS, including singlet oxygen and one-electron oxidant, but detailed consideration of them is out of scope of this work [30]. DNA damage in the form of oxidatively modified bases is repaired by base excision repair pathway, consisting of DNA glycosylases, AP-endonucleases, phosphodiesterases, DNA polymerases and DNA ligases (see [31] for review).

As mentioned, differentiating cells extensively transcribe genes, which products are essential for their rapid development, so coping with DNA damage stalling RNA polymerase is equally essential. The cells have evolved transcription-coupled repair, which deals with DNA damage more effectively than genome-wide repair (see [32] for review). In the present work we applied the comet assay, which provides information on actual DNA damage level in single cells. This actual level is usually a resultant of the action of DNA-damaging agents and DNA repair systems, both genome-wide and transcription-coupled. We observed the protective action of both DEX and 1,25-D3 against oxidative DNA damage and we can speculate that several mechanisms may be involved in this protective effects. First, either or both substance may chemically inactivate an oxidant, in our case TBH. Second, it or they may stimulate the repair of lesions induced by TBH. Third, DEX and 1,25-D3 may modulate DDR function of RUNX2, modulating observed DNA damage level. All these hypotheses may be verified in a perspective study.

We showed that DEX and 1,25-D3, which are considered as modulators of RUNX2 activity, significantly changed the process of differentiation and influenced DNA damage response in differentiation osteoblast. Previously, we showed that they change DDR also in non-stimulated osteoblasts [33]. However, the present experiment does not allow for drawing a definite conclusion as we signaled that there were controversial results on the final effect of DEX and 1,25-D3 on RUNX2 activity in differentiating osteoblasts. Let us take a closer look at this issue.

Dexamethasone, a synthetic glucocorticosteroid analogue, may act bilaterally on osteoblasts differentiation process, depending on its dose and incubation time [34]. The exact nature of such bidirectional character of DEX is not clear and it is hard to find the borderline concentration of DEX between its stimulating and inhibitory actions.

The effect of DEX on osteogenesis may be mediated at least partially by RUNX2 but results on its mechanism of action are divergent. One study reported enhanced transcriptional activity of RUNX2 after DEX treatment, while other showed a synergistic effect of DEX and RUNX2 on osteocalcin and bone sialoprotein gene expression, alkaline phosphatase activity, and biological mineral deposition in primary dermal fibroblasts [35]. Other group demonstrated that DEX-mediated RUNX2 inhibition was not attributed to its transcriptional activity, but rather to physical interaction between RUNX2 and glucocorticoid receptor, and 1 nM DEX was sufficient to inhibit RUNX2 [36].

It was suggested that inhibitory effect of DEX on osteogenesis may be associated with apoptosis and G1 phase arrest. This effect was diminished when p53 was inhibited by p53 RNA interference [37]. Therefore, a complex, mutual interaction between DEX, RUNX2 and proteins of DDR might occur in our experiment.

1,25-Dihydroxyvitamin D3 is the most biologically active form of vitamin D3, involved in cell growth and differentiation, calcium homeostasis, bone metabolism and immune system modulation. These roles are mediated through a vitamin D receptor (VDR) [38]. 1,25-D3 plays an important role in transcriptional regulation of multiple osteogenesis-related genes, including RUNX2 upstream regulators [39]. Its high stimulatory effect on ALP expression in osteoblasts was also shown [40]. Moreover, RUNX2 was reported to directly interact with VDR in promoter regions of target genes [41]. It was demonstrated that RUNX2 expression was down-regulated by 1,25-D3 in MC3T3-E1 and ROS 17/2.8 cells [42,43].

1,25-D3 may play a role in the regulation of oxidative stress and DNA damage response in osteoblasts. Since apoptosis plays a key role in bone remodeling, it may link both processes, determining cell fate. It has been shown that physiological concentrations of 1,25-D3 may protect osteoblasts against apoptosis [44,45]. Moreover, 1,25-D3 exposure reduced the level of γH2AX and ATM-S1981P expression following H_2_O_2_ treatment [46]. Moreover, the level of intracellular ROS was also diminished in that experiment.

In summary, the results we obtained suggest important role of RUNX2-modifiers in DNA damage response induced by oxidative stress in differentiating osteoblasts, but the mechanisms underlying this role is unknown. It should be stressed that we do not have any direct evidence on the involvement of RUNX2 in DDR in our experiment. The attitude we employed—chemical modification of RUNX2 activity is somehow confusing, as the modificators we used, dexamethasone and 1,25-dihydroxyvitamin D3, have no unequivocal impact on RUNX2 and they may themselves modulate oxidative stress and DDR and a genetic approach, e.g., using of miRNA or shRNA, would be likely more suitable. However, our work demonstrate a complexity and importance of relationships between oxidative stress, DNA damage response and supplementation with some modifiers of RUNX2 activity during osteoblasts differentiation, which may be important for control of bone formation and prevention of bone diseases.

## 3. Experimental Section

### 3.1. Reagents

2',7'-Dichlorodihydrofluorescein diacetate (DCFH-DA), *tert*-butyl hydroperoxide 98% (Luperox, TBH), l-Ascorbic acid 2-phosphate sesquimagnesium salt hydrate (l-AA), β-glycerophosphate disodium salt hydrate (β-GP), 1,25-dihydroxyvitamin D3 (1,25-D3), dexamethasone (DEX), DAPI (4',6-diamidino-2-phenylindole), low and normal melting point agarose, phosphate saline buffer (PBS) were obtained from Sigma-Aldrich (St. Louis, MO, USA). Cell culture reagents: α Minimum Essential Medium (α-MEM), l-Glutamine, antibiotics (Pen Strep), fetal bovine serum (FBS) and 0.05% Trypsin-EDTA were obtained from Life Technologies (Thermo Fisher Scientific, Waltham, MA, USA). Alkaline phosphatase (ALP) activity colorimetric assay kit was from Bio Vision Inc. (Milpitas, CA, USA).

### 3.2. Cell Culture

The mouse preosteoblast cell line, MC3T3-E1, was provided by the European Collection of Cell Cultures through the Sigma-Aldrich. Cells were grown in α-MEM supplemented with 10% FBS, 2 mM l-Glutamine and 1% antibiotics. Cultures were incubated at 37 °C in the presence of 5% CO_2_ in a humidified atmosphere. The complete growth medium was replaced every 2–3 days and confluent cells were subcultured through trypsinization.

### 3.3. Induction of Osteoblast Differentiation

To study cell differentiation of preosteoblasts, the cells were harvested and seeded on the 96-well or 24-well plates and were allowed to reach 80% confluence. Differentiation was then induced through the addition of 50 μg/mL l-AA and 10 mM β-GP to the growth medium, in the presence or absence of tested compounds—RUNX2 inhibitors, 1,25-D3 at 10 and 50 nm and DEX at 1 and 5 μM. This was considered the day 0 of the experiment. Differentiation medium with or without RUNX2 inhibitors was subsequently replaced every 48 h, with sample analysis or preparation for qualitative assessment on day 3, 6, 12, and 24.

### 3.4. Alkaline Phosphatase Activity Assay

The commercial Alkaline Phosphatase Activity Colorimetric Assay Kit (Sigma-Aldrich, St. Louis, MO, USA) was used, which employs *p*-nitrophenyl phosphate as an ALP substrate which turns yellow (λ_max_ = 405 nm) when dephosphorylated by ALP. The change of color was detected in a PowerWave XS plate reader (Bio-Tek Instruments, Winooski, VT, USA). Each assessment was performed in triplicate.

### 3.5. Induction of Oxidative Stress and Its Assessment

An aliquot of the cells was taken at time 0 and on 3, 6, 12 and 24 days and the cells were challenged for 10 min with 50 or 100 μM TBH at 37 °C. Then, TBH was washed out and the levels of ROS was determined. We considered ROS level as a metric of oxidative stress. It was determined on the basis of oxidative conversion of non-fluorescent DCFH-DA to fluorescent 2',7'-dichlorofluorescein (DCF) [47]. The cells were seeded into 96-well black plates at a density of 5000/well and then incubated with TBH as described above. The supernatant was then discarded and replaced with 10 μM DCFH-DA and cells were cultured in the dark for 45 min. The fluorescence intensity was measured in a Thermo Scientific plate reader, model Fluoroscan Ascent FL (Thermo-Scientific, Waltham, MA, USA). Each measurement was performed in triplicate.

### 3.6. Comet Assay for DNA Damage

The extent of DNA damage was evaluated by the comet assay, which was performed under alkaline conditions according to the procedure of Singh *et al.* [48] with modification [49] as previously described [50]. A freshly prepared suspension of osteoblasts in 0.75% low melting point agarose dissolved in phosphate buffered saline was cast onto microscope slides precoated with 0.5% normal melting agarose. The cells were then lysed for 1 h at 4 °C in a buffer consisting of 2.5 M NaCl, 100 mM EDTA, 1% Triton X-100, 10 mM Tris, pH 10. After the lysis, DNA was allowed to unwind for 40 min in electrophoretic solution consisting of 300 mM NaOH, 1 mM EDTA, pH > 13. Electrophoresis was conducted at 4 °C for 30 min at electric field strength 0.73 V/cm (30 mA). The slides were then neutralized with 0.4 M Tris, pH 7.5, stained with 2 μg/mL DAPI and covered with cover slips.

The slides were examined at 200× magnification in an Eclipse fluorescence microscope (Nikon, Tokyo, Japan) attached to a COHU 4910 video camera (Cohu, Inc., San Diego, CA, USA) equipped with a UV filter block consist an excitation filter (359 nm) and barrier filter (461 nm) and connected to a personal computer-based image analysis system, Lucia-Comet v. 4.51 (Laboratory Imaging, Prague, Czech Republic). Fifty images were randomly selected from each sample and the comet tail DNA was measured. Two parallel tests with aliquots of the same sample of cells were performed for a total of 100 cells. Each experiment was repeated two times. Percentage of DNA in the tail (% tail DNA) was analyzed. It is positively correlated with the level of DNA breakage or/and alkali labile sites in the cell and is negatively correlated with the level of DNA crosslinks [51,52]. The mean value of the % tail DNA in a particular sample was taken as an index of DNA damage in this sample.

### 3.7. Data Analysis

The values in this study were expressed as mean ± SEM from three experiments, *i.e.*, the data from three experiments were pooled and the statistical parameters were calculated. The Mann-Whitney test was used to determine differences between samples with distributions departing from normality. The differences between samples with the normal distribution were evaluated by applying the Student’s *t*-test. Data analysis was performed using SigmaStat software (v. 3.0.0, SPSS, Chicago, IL, USA)

## 4. Conclusions 

We conclude that differentiation of osteoblasts is associated with oxidative stress, which may cause DNA damage, which, in turn, induces DNA repair. This response is modulated by modifiers of the RUNX2 protein, a master regulation of bone formation, which indirectly suggests that RUNX2 might be involved in cellular response to DNA damage evoked by oxidative stress.
